# The CAESAR project for *in silico *models for the REACH legislation

**DOI:** 10.1186/1752-153X-4-S1-I1

**Published:** 2010-07-29

**Authors:** Emilio Benfenati

**Affiliations:** 1 Istituto di Ricerche Farmacologiche Mario Negri, Via La Masa 19, 20156, Milano, Italy

## CAESAR and the REACH legislation

REACH is the new European legislation for the safe use of chemicals [1]. It is an enormous initiative, which requires information for all chemicals which are currently on the market in Europe in quantities above one tonne per year. A huge amount of data on each compound is required, and alternative methods to direct experimentation such as *in silico *models are listed as possible sources of this information. *In silico *models are those aiming to predict the properties of a chemical compound on the basis of its chemical structure. However, this poses the problem of the correct use of these methods.

CAESAR is a unique project funded by the European Commission, dedicated to developing *in silico *models specifically designed for the REACH legislation. The legislation has shaped each step of the modeling process.

There are thousands of *in silico *models which address new descriptors, new algorithms, or adopt a new method to predict a certain endpoint. Indeed, activities in the *in silico *area exploded in a multitude of aspects. However, typical *in silico *models are not suitable for regulatory purposes, as they have not taken into account one or more factors essential for validation, quality assurance, or for a specific application of a given compound.

The typical approach for *in silico *modelling is to develop a model and *then *propose its use. However, it is clear that this is a generic approach, not addressing a specific application.

## The REACH requirements

To propose an *in silico *model for REACH, we must consider the use of the resulting data within REACH. REACH promotes the use of all data, but it contains clear guidelines which should be adopted to ensure good data quality. In case of *in vivo *data, specific official protocols are listed to get high quality data, following OECD or USEPA procedures. Similarly, when *in vitro *data are used, the preferred procedures are identified. When data have been produced without reference to the official guidelines, their quality should be evaluated.

Another important point is that for some endpoints no suitable protocol exists yet.

In case of *in silico *models and in particular QSAR models which use quantitative structure-activity relationships, REACH says that these should be scientifically valid. We believe that the CAESAR models are scientifically valid, and we validated them according to a series of procedures including internal and external validation.

In addition, according to REACH these methods should be used within their applicability domain. We developed a number of diverse, innovative, specific tools to directly assess this. We identified a series of chemical groups/classes for which the model does not offer optimal performance to be used as a basis for warning. Furthermore, the models automatically highlight if some reasons of concern appear for the ideal use of the model, e.g. boundaries of the descriptor space. Finally, the model shows the predicted value/category, the experimental one, and the error of the six most similar compounds, in relation to the target compound for prediction. Thus, the user has a clear, immediate appreciation whether similar compounds were included developing the models and how accurate were their predictions. A similarity score between compounds is also provided, to guide the user.

Another aspect mentioned by REACH is that models should be appropriate for the targets of REACH, such as classification and labelling, prioritisation and risk assessment. Indeed, we evaluated the nature of the output of our models, because in some cases continuous values are required, for risk assessment, while in other cases a categorical output is needed, for classification and labelling.

Furthermore, the values produced from QSAR models should fit as much as possible to the common process of using data. As we said, data (and most typically we refer to *in vivo *data) should be produced according to good procedures and of checked quality. Thus, also in the process of model building used within CEASAR, we dedicated the highest attention of the quality of the data used as input for our models. The data on chemical compounds are at the basis of any *in silico *model. Within CAESAR we spent about one year checking data, before starting the modelling procedure. We identified errors in the existing data collections, in some cases quite numerous, in other cases minor. We notice that errors should be checked always for regulatory purposes, not only when building up QSAR models.

Furthermore, we notice that different thresholds exist depending on the legislation, and this also calls for specific models.

There are other important parameters to be carefully evaluated when developing models for regulatory purposes. In particular, false negatives should be minimised. False negatives are the predictions that a given compound is toxic, while in reality it has toxic potential. It is clear that this error has a different impact compared to false positives. Regulators want to avoid false negatives, while false positive are not so critical.

This requires special adapdations and checks to be incorporated in the models, which are typically not done when a model is developed or evaluated for other purposes. Indeed, most typical QSAR models are evaluated using squared errors and differentiation between false positives and negatives are therefore lost.

CAESAR addressed all these critical factors and goes beyond the question of scientific quality, which have been established and discussed eg. in the OECD principles for QSAR models [2].

Dealing with tools for regulatory purposes, another important point is the reproducibility of the method or model. It is obvious that, in the case of a QSAR model providing different results with different users, the value of such a model is seriously questionable. We should mention that some *in silico *models give different results, based on expert experience. We believe that for regulatory purposes the possibility to get reproducible results is an advantage. Indeed, if different results can be obtained, for instance, by the industrial or regulatory user, or in different countries, this complicates the assessment of the results, and needs a separate evaluation of the results.

Vice versa, for other purposes, it can be accepted that the user skill plays a role. For instance, there are complex programs which require careful optimisation of certain parameters, such as three-dimensional conformation. Experts are requested to run these programs properly. The use of these complex tools is more suitable within industry for their aims, such as drug design, where the role of the expert is usually fundamental.

## Models for stakeholders

Within CAESAR we dedicated special attention to the users and the access of the models. Thus, we preferred to develop models which are simple to used. We dedicated special studies to facilitate the user. A graphical interface guides the user through three easy steps. CAESAR is intuitive and in this it has been preferred within a training course when different models have been presented. The particular application of REACH needs to keep into account the stakeholders. This means that the issues to be evaluated are not only theoretical but practical. If a certain model requires special experience, this is a barrier.

We preferred to follow an approach such as that used by the US EPA. The same easy model is available to all stakeholders: industry and regulators. Thus, the same rules are known, the model is transparent and reproducible.

The CAESAR models can be used to process a large number of chemicals in batch. This fact also increases the versatility and usability, and thus the impact of the models. Indeed, the CAESAR models can be used to screen a large number of compounds, for instance for prioritisation, which is a target mentioned by REACH, and particularly useful for regulatory bodies such as the European Chemicals Agency. Recently, it was found that the number of chemicals which potentially must be evaluated, on the basis of the preregistration of the chemical substances, is much higher than expected. This higher number may mean that the actual number of substances checked may be low. From this perspective, the availability of tools such as those developed by CAESAR is highly beneficial for regulators, as they can easily and rapidly process a large number of compounds.

Thus the potential impact of CAESAR's model, if adopted by regulators for screening purposes, is that regulators could easily process all compounds, and identify critical issues. This can improve the evaluation of the chemicals in the European market, and eventually minimise the possibility of toxic compounds making their way onto the market.

Furthermore, the CAESAR models are free. Indeed, some of the addressed endpoints are expensive to study experimentally. There have been critical comments that REACH will impose a high cost on industry, but the use of CAESAR's model can solve this issue, for the specific endpoints we studied.

Furthermore, as we explained, the use of the models can be pro-active, to anticipate future potential problems related to certain chemical structures. This is a further useful possibility of larger use, and thus models can have an impact on industry, which can run the models when planning new compounds, and assess in advance if a certain structure may present critical aspects. As a result, industry will have a powerful tool to produce safer chemicals, which is the final target of REACH.

Since the models are free, they can be easily used by non-governmental organisations interested in safety assessment.

The CAESAR models are available on the internet http://www.caesar-project.eu. This facilitates their access on a worldwide basis. We will describe more in detail the software used to implement selected models developed within CAESAR in the five papers of this issue.

## The five CAESAR models

CAESAR developed new QSAR models specific for REACH for these endpoints:

• bioconcentration in fish,

• skin sensitisation,

• mutagenicity,

• carcinogenicity,

• developmental toxicity.

Many tens of different models have been developed for each endpoint, and we implemented some of the best ones in the internet platform. The papers in this issue describe some of these best models, and their implementation on the internet.

All of them match the criteria described above. We do not consider these models as the ultimate solution, but we believe they represent a major improvement in the current scenario for the reasons described, and because the results of these models have been assessed as being as good as or more frequently better than those obtained with other models.

We believe that in some cases the models can replace the current method, and in all cases the information obtained from the model is a valuable supplement to other sources of data. This depends on the endpoint and on the chemical.

## Competing interests

The author declares that they have no competing interests.
